# Cue Utilization and Cognitive Load in Novel Task Performance

**DOI:** 10.3389/fpsyg.2016.00435

**Published:** 2016-03-29

**Authors:** Sue Brouwers, Mark W. Wiggins, William Helton, David O’Hare, Barbara Griffin

**Affiliations:** ^1^Macquarie UniversitySydney, NSW, Australia; ^2^University of CanterburyChristchurch, New Zealand; ^3^University of OtagoDunedin, New Zealand

**Keywords:** cue utilization, cognitive resources, cognitive load, workload

## Abstract

This study was designed to examine whether differences in cue utilization were associated with differences in performance during a novel, simulated rail control task, and whether these differences reflected a reduction in cognitive load. Two experiments were conducted, the first of which involved the completion of a 20-min rail control simulation that required participants to re-route trains that periodically required a diversion. Participants with a greater level of cue utilization recorded a consistently greater response latency, consistent with a strategy that maintained accuracy, but reduced the demands on cognitive resources. In the second experiment, participants completed the rail task, during which a concurrent, secondary task was introduced. The results revealed an interaction, whereby participants with lesser levels of cue utilization recorded an increase in response latency that exceeded the response latency recorded for participants with greater levels of cue utilization. The relative consistency of response latencies for participants with greater levels of cue utilization, across all blocks, despite the imposition of a secondary task, suggested that those participants with greater levels of cue utilization had adopted a strategy that was effectively minimizing the impact of additional sources of cognitive load on their performance.

## Introduction

Skilled performance across a range of domains of practice is characterized by accurate and rapid responses, often in dynamic and complex situations (Salthouse, 1991; Ericsson and Lehmann, 1996; Beilock et al., 2004). This is attributed to specialized routines or associations that have been established through repeated application across a variety of settings (Klein, 2011). These highly specialized associations, representative of situation-specific relationships between environmental features and events or objects, are often referred to as cues (Brunswik, 1955; Klein et al., 1986; Wiggins, 2014), and their activation and retrieval from long-term memory has the advantage of imposing relatively fewer demands on working memory resources (Norman and Shallice, 1986; Chung and Byrne, 2008; Evans, 2008).

Differences in the rate at which individuals acquire skills have been attributed to various factors, including cognitive style (Cegarra and Hoc, 2006), motivation and self-regulation (Zimmerman, 2002, 2008), cognitive ability and intelligence (Ackerman, 1986, 2007; Ackerman and Beier, 2007), personality (Singer and Janelle, 1999; Simonton, 2008), and a range of general intrinsic abilities (Thompson et al., 1993; Simonton, 2007, 2008). However, in some environments, the acquisition of skilled performance is also characterized by the capacity to rapidly and accurately extract and utilize meaningful information from features in the environment (Abernethy, 1987, 1990; Bellenkes et al., 1997), thereby enabling the discrimination of relevant from less relevant cues (Weiss and Shanteau, 2003).

Evidence to support the utilization of cues in skill acquisition can be drawn from investigations involving fast ball sports, in which skilled performers anticipate the trajectory of a target by restricting their attention to a limited number of highly predictive features (Müller and Abernethy, 2012; Moore and Müller, 2014). These features include the wrist angle of the bowling arm in cricket (e.g., Müller et al., 2006) and the location of the ball just prior to contact with the racket following a tennis serve (e.g., Jackson and Mogan, 2007).

The rapid identification of a limited number of predictive features has a range of benefits for skill acquisition, including a reduction in the demands on cognitive load and an improvement in the rate of skill acquisition. For example, Perry et al. (2013) were able to demonstrate improvements in performance amongst novice fire fighters by restricting their information acquisition only to those features that were sourced by skilled fire commanders. Although the discrimination between relevant and less relevant features was contrived in this case, it suggests that a general capability to identify a limited number of highly predictive features may explain differences in rates of skill acquisition during unimpeded learning tasks.

Wiggins et al. (2014) demonstrated a relationship between a general capacity for cue utilization and skill acquisition in experiments involving learning to land an aircraft and learning to operate a line-of-sight Unmanned Aerial Vehicle (UAV). Using the situation judgment test EXPERTise (1.0) (Wiggins et al., 2010) to provide a composite assessment of cue-utilization, greater levels of cue utilization were associated with improved accuracy in landing the aircraft following four trials, and with fewer trials to reach criterion in learning to take-off and land a UAV. These improvements in performance occurred in the absence of any formal instruction. However, it was unclear whether these improvements were a consequence of participants’ capacity to quickly establish feature-event relationships in the form of cues, and/or whether this capacity reduced the demands on cognitive load, thereby enabling learners to reinforce, revise, or refine the relationships that had been acquired during the initial stages of skill acquisition. The aim of the present study was to investigate, in the context of a low workload, novel task, whether differences in a general capacity for cue utilization are evident in performance, and whether these differences reflect differences in the management of cognitive load.

Where there are multiple courses of action to achieve an outcome, humans will normally select strategies that are associated with the least cognitive effort (Kool et al., 2010). This is referred to as Hull’s (1943) law of less work, whereby mental effort is regarded as an aversive stimulus. Therefore, in responding to a novel task, the capacity to identify quickly the strategy of least cognitive effort, while maintaining performance, represents an adaptive approach that conserves cognitive resources.

When exposed to a novel task, participants with a relatively greater capacity for cue utilization would normally be expected to quickly identify key features associated with the performance of a task which, in turn, reduces cognitive load, thereby providing an increased capacity for skill acquisition (Wiggins, 2015). The present study comprised two experiments in the context of rail control, in which participants were asked to respond to misrouted trains. Importantly, however, participants had seven seconds in which to formulate an assessment, and this represented a key feature that, when identified, would enable participants to minimize the cognitive load imposed by the task.

Consistent with actual rail control, the experimental task was semi-automated, so that it constituted a low workload environment that demanded sustained attention to identify only those trains that required an intervention. Drawing on Resource Theory (Helton et al., 2005; Helton and Warm, 2008), sustained attention to a task is presumed to impose a cognitive demand on information processing, leading to vigilance decrements that include an increase in errors and/or response latency across an extended exposure. Therefore, there was an implicit incentive for participants to adopt a strategy that would reduce cognitive load. In the present study, Experiment 1 examined the relationship between cue utilization and performance on a simulated rail control task over a 20-min period of watch. Experiment 2 involved the imposition of a concurrent secondary task that was intended to, more explicitly, increase cognitive load.

## Experiment 1

Experiment 1 was designed to examine the relationship between a composite measure of cue utilization, and performance on a simulated rail-monitoring task that required participants to correctly reroute trains that were periodically misrouted. Trains traveled at a consistent and relatively slow rate, and only trains on incorrect routes required a response.

The simulated rail task was designed to incorporate specific elements of ecological validity, including the requirement to monitor multiple rail lines simultaneously, the requirement to intervene periodically, and the requirement to intervene within a specified period of time (Lenior, 1993; Neerincx and de Greef, 1998; Ho et al., 2002; Farrington-Darby et al., 2006). Aside from the adjustment of train routes, which is a fundamental task performed by real-world rail controllers (Neerincx and de Greef, 1998), the movement of trains to and from different directions was also captured in the simulation interface. To account for the demands of experimental control, higher level features of real railway control systems such as the connection of track elements to a network (Berkenkötter and Hannemann, 2006) and the determination/ communication of critical incidents (Farrington-Darby et al., 2006) were not incorporated in the simulation task. Given the requirement for sustained attention, the rail-monitoring task continued over a 20-min period of watch. A 20-min period of watch was selected because previous research has found evidence for an observable vigilance decrement within that period of time (Temple et al., 2000; Rose et al., 2002; Helton et al., 2005; Small et al., 2014).

Based on the proposition that a propensity for cue acquisition enables the rapid identification of feature-event relationships, the performance of those participants with relatively greater levels of cue acquisition would, over a consistent period of exposure to a novel task, be impacted to a relatively lesser extent by the imposition of cognitive load. Since sustained attention is associated with increases in cognitive load (Helton et al., 2005; Helton and Warm, 2008), it was anticipated that, while all participants would experience a vigilance decrement during the latter part of the vigil, participants with greater levels of cue utilization would experience the least increases in response latency coincident with the increase in cognitive load. Specifically, it was hypothesized that: (a) a main effect would be evident for response latency, in which all participants would experience an increase in response latency during the latter stages of the vigil, and (b) that an interaction would be evident, wherein participants with lesser levels of cue utilization would record a greater increase in mean response latency between the first and last 5-min blocks for accurate responses to misrouted trains, in comparison to participants with greater levels of cue utilization.

### Method

#### Participants

A total of 58 first and second year university students (41 females and 17 males) were recruited for the study, each of whom received course credit in return for their participation. Participants ranged in age from 18 to 22 years (*M* = 19.26, *SD* = 1.35). The inclusion criteria comprised existing motor vehicle drivers who had not been exposed to train control operations, and who were aged between 18 and 22 years. Utilizing a cohort of 18 to 22 year old drivers enabled comparative assessments of cue utilization, controlling to a limited extent, exposure to driving.

#### Instruments

Participants were asked to indicate their age, gender, months of driving experience, daily driving frequency, and their experience in rail control. Cue utilization was assessed using the Expert Skills Evaluation (EXPERTise 1.0) (Wiggins et al., 2010) situation judgment test.

##### EXPERTise 1.0

EXPERTise 1.0 consists of experimental tasks that have been individually and collectively associated with differences in performance at an operational level (Loveday et al., 2013a,b,c, 2014). Consistent with the notion that there are individual differences in populations for cue utilization, the driving version of EXPERTise was selected, as it assesses the acquisition of cues in a specific cohort and at a specific point in time, and it is a context with which participants would be familiar (Wiggins et al., 2014). Tasks in the EXPERTise driving battery include a paired association task, a feature discrimination task, a feature identification task and an information acquisition task.

In the *Paired Association* task, participants are presented with two feature-event/object terms. Over a total of 30 trials, each two terms are displayed, adjacent to one another for 1500 milliseconds. After each pair is displayed, participants indicate the extent to which the two terms are related on a 6-point Likert scale (from 1 = “Extremely unrelated” to 6 = “Extremely related”). Examples include the related terms ‘heavy traffic’ (feature) and ‘short-cut’ (event) and relatively less related terms ‘traffic-light’ (feature) with ‘free-way’ (object). Higher levels of cue utilization are associated with a greater variance in the perceived relatedness of terms (Ackerman and Rathburn, 1984; Schvaneveldt et al., 2001; Morrison et al., 2013).

In the *Feature Discrimination* task, participants are presented with a short, written description of a single scenario (i.e., “You are lost in an unfamiliar area. You find yourself in a quiet suburban area, and must find your way to a large shopping center located on a main road. You can see heavier traffic on a main road ahead and high-rise buildings are in the distance...”). Participants are then asked to make a decision based on their typical response in this scenario (i.e., drive in the direction of heavier traffic, or drive toward high-rise housing, and so on). Following their decision, participants are presented with a list of fourteen features and, using a 10-point Likert scale (from 1 = “Not important at all” to 10 = “Extremely important”), are asked to rate these features based on their perceived relevance to his/her decision. Greater levels of cue utilization are associated with higher variances within the feature-relevance ratings (Weiss and Shanteau, 2003; Pauley et al., 2009).

The *Feature Identification* task involves the extraction of key information from an array or scene. Participants are presented with a familiar driving scene (i.e., an image of a road as viewed from the driver’s seat of a car) and are directed to identify a road hazard as quickly as possible (i.e., a ball positioned in the road ahead). The position of the ball changes over trials. A lower mean reaction time is associated with greater levels of cue utilization (Schyns, 1998; Schriver et al., 2008; Loveday et al., 2014).

Finally, the *Information Acquisition* task presents participants with a way-finding scenario that requires a choice between three different driving routes. Accompanying the scenario instructions is a drop-down menu with 24 options (feature-cues), which are category-labeled (e.g., ‘distance’, ‘weather conditions’) and upon selection, provide participants with information pertaining to the distance, tolls, road works, weather conditions, traffic congestion, speed limit, and the number of lanes for each route. Participants are given one minute to select information prior to making a response. This task assesses the capacity to acquire feature cues from the environment in a prioritized and non-linear pattern (Wiggins and O’Hare, 1995; Wiggins et al., 2002). Individuals with lesser levels of cue utilization are more likely to select information in the sequence in which it is presented (e.g., from left to right as they appear on the display screen). Greater levels of cue utilization are associated with a relatively lower ratio of pairs of information screens accessed in the sequence in which they are presented, against the total frequency of pairs of information screens selected.

The criterion validity of EXPERTise (1.0) has been established in a number of different domains in which typologies formed on the basis of EXPERTise performance differentiated workplace-related performance (Loveday et al., 2013a,b,c). The test–retest reliability (*κ* = 0.59, *p* < 0.05) has been demonstrated with power control operators at six-monthly intervals (Loveday et al., 2013a). In the present study, restricting the age of participants (18–22 years) controlled for exposure to driving experience. This ensured that any differences in cue utilization would be unlikely to result from differences in driving experience. Overall, participants had accumulated a mean of 39 months of driving experience (*SD* = 15.82 months).

##### Rail control task

**FIGURE 1:**
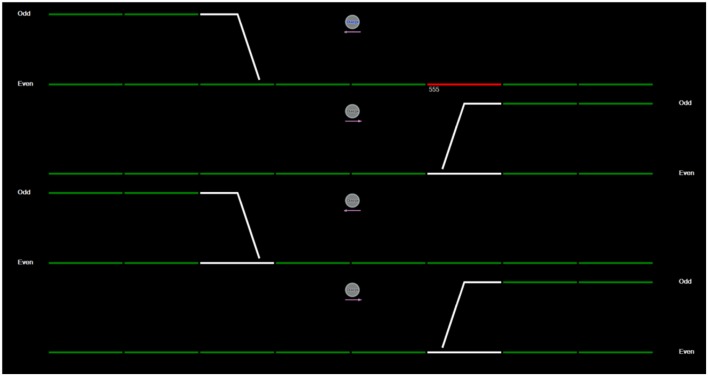
**The simulated train-control display as viewed by participants.** The four long, horizontal green lines represent railway tracks. The white portions on each track are the intersection lines, which are controlled by an interlocking switch labeled, “Change”. This switch is depicted by a small circle icon, located above each track. If a participant selects the “Change” icon, any train traveling on the track beneath it, will be diverted onto the intersecting line.

A simulated train control task was used as a novel, low workload context for the present study. In this task, a computer screen depicts a simulated, simplified train control display (see **Figure 1**).

Within the train task display, four long, horizontal green lines represent railway tracks (See **Figure 1**). Each track incorporates an intersection (depicted by white portions on the track), which is controlled by an interlocking switch labeled, “Change”. This switch is depicted by a small circle icon, located above each track. If a user selects the “Change” icon, (with a computer mouse), any train traveling on the connected track will be diverted onto the intersecting line.

A train is depicted by a red horizontal bar that appears at one end of a train line, and travels across the display. Each train has a three-digit number assigned as either odd or even (e.g., 888, 333). Each train line and its associated branch line also have an assigned label: Odd or Even. As the train appears onto the screen, a green line depicts the programmed route of the train. The participant’s task is to ensure that trains run along the correct train lines (even-numbered trains run along even lines and odd-numbered trains along odd lines). Periodically, programmed routes will appear that are inconsistent with the train’s number so that, for example, an even numbered train is programmed to take a route that is labeled ‘odd’. To correct the programmed route of the train, participants must select the “Change” icon which will re-route the train.

Once a train appears on the computer screen, participants have seven seconds in which to decide whether or not to reroute a train. All trains travel at the same speed and trains appear within 5–30 s of each other. Therefore, the screen may display a static image of train lines (without any trains) for up to 30 s before another train appears. A total of 67 trains appear on the four rail lines over the course of 20-min, half of which are not required to be re-routed. Data recorded from this task included response latency (in milliseconds, from the initial appearance of a train, to the selection of the “Change” icon) and the accuracy of responses (whether trains were diverted when required).

##### Cognitive ability

The Raven’s Standard Progressive Matrices cognitive test (SPM; Raven et al., 1998, 2000) was included as a measure of cognitive ability. The SPM broadly assesses general problem solving ability or fluid intelligence by measuring the capacity to recognize and process patterns of spatial information (Raven et al., 2000; Kaplan and Saccuzzo, 2008). Cognitive ability encompasses constructs that include processing speed and working memory capacity (Conway et al., 2002) that can influence performance in attention-demanding tasks (Kane and Engle, 2003). In the present study, the SPM was included as a means of establishing whether cognitive ability was related to performance scores in the rail task. The SPM short version (10-min timed) was used (see Caffarra et al., 2003; Austin, 2005; Moutafi et al., 2006; Jaeggi et al., 2011). Cognitive ability scores reflected the total number of correct SPM responses.

##### The group embedded figures test

The Group Embedded Figures Test (GEFT: Witkin et al., 1971, 2002; Oltman et al., 2003) is a perceptual test that assesses an individual’s field dependence-independence. According to Witkin (1976), Field Independence–Dependence is a cognitive style that represents the extent to which an individual can overcome the influence of irrelevant background elements when attending to a task. Individuals who exhibit higher levels of field independence more easily overcome background elements in formulating judgments. The GEFT requires the test taker to identify and trace simple forms (i.e., shapes) that are embedded within more complex forms. The Embedded Figures Test has been linked to the capacity to perceive hazards, recognize faults and formulate mental representations of problems (Vessey and Galletta, 1991; Elander et al., 1993; Leach and Morris, 1998). The GEFT was included in the present study to ascertain whether rail task responses were related to cognitive style. Test–retest reliability coefficients for the GEFT range from 0.79 to 0.92 over multiple time intervals of up to 3 years (Kepner and Neimark, 1984; Witkin et al., 2002).

#### Procedure

Following approval of the study by the Macquarie University Human Research Ethics Committee, participants were recruited and tested individually in 90-min sessions. After completing an on-line demographic questionnaire, a computer prompt directed the participants through the four EXPERTise tasks. Standardized instructions for the rail task were then provided verbally. This included the verbal instruction, “the aim of this task is to ensure that each train is on its correct track”. No information or direction was provided in relation to the speed or pace of the task (i.e., participants were not told that they had several seconds of decision-time available or that they could or should respond in either an immediate or delayed manner). After a 5-min trial to orient the participants to the task, the, 20-min experimental trial commenced. Participants then completed paper-and-pencil versions of the SPM and GEFT. Instructions for these tests were provided to participants verbally and through written directions, according to the test instruction manuals.

### Results

#### Preliminary Analysis

##### Rail task performance scores

Response latency for correct responses in the rail task comprised the primary dependent variable. Latencies were calculated from the initial appearance of a train to the selection of the ‘change’ icon where appropriate. Errors occurred when a train was re-routed from its correct path (a false alarm) or was not re-routed when required (a miss). The number of errors made by participants ranged from zero to five, with a median of one, and resulted in a floor effect, with 64% of the entire sample recording either zero or a single error during exposure to the 67 trains. A Spearman’s rank-ordered, non-parametric correlation between the number of errors committed in the rail task and mean response latencies was not statistically significant. The relationship between error frequency and interval, examined using a chi-square test of independence, failed to reveal any statistically significant variation in the distribution of errors across the four time intervals, χ^2^(3, 58) = 5.026, *p* = 0.17. Taken together, these results suggest that a speed-accuracy trade-off was not necessary to undertake the task successfully.

Since the task was 20-min in duration, the mean response latencies (for correct responses) were calculated across four, 5-min intervals, and these four variables comprised the dependent variables in subsequent analyses. Nineteen trains appeared within the first block, nine of which required re-routing. In the second block, 16 trains appeared, eight of which required re-routing. In the third block, 15 trains appeared, seven of which required re-routing, and in the final time block 17 trains appeared, of which nine required re-routing.

##### Cognitive ability and cognitive style

Scores on the SPM were normally distributed and not significantly correlated with mean response latencies for any of the four blocks of trials (–0.04 ≤ *r* ≤ –0.15, *p* > 0.05). As GEFT (cognitive style) scores were negatively skewed, a square root transformation with reflection was applied to normalize the data. Subsequent Pearson’s correlations failed to reveal any statistically significant associations between GEFT scores and mean response latencies across any of the four blocks of trials (–0.03 ≤ *r* ≤ 0.22, *p* > 0.05).

##### Cue utilization typologies

Prior to analysis, it was necessary to identify the cue utilization typologies that corresponded to relatively greater or lesser levels of cue utilization (Loveday et al., 2013a,b; Wiggins et al., 2014). Consistent with the standard approach to EXPERTise data, *z* scores were calculated for each task, with those corresponding to the Information Acquisition and Feature Identification tasks reversed so that for all four tasks, higher *z* scores represented greater levels of cue utilization. A cluster analysis identified two groups with centroids corresponding to higher variance in the Paired Association and Feature Discrimination tasks, lower response latency in the Feature Identification task (reversed *z* score), and a lower ratio of sequential selections in the Information Acquisition task (reversed *z* score). The cluster analysis classified 34 participants in the lesser cue utilization typology and 24 participants in the greater cue utilization typology (**Table 1**).

**Table 1:** Cluster centroids for the EXPERTise task scores.

	Cluster 1 (*n* = 34)	Cluster 2 (*n* = 24)
Paired association	–0.60	0.86
Feature discrimination	–0.52	0.74
Feature identification	–0.12	0.17
Information acquisition	–0.40	0.57

##### Driving experience and cue utilization

To examine whether differences in cue utilization resulted from differences in participants’ length of driving experience, a one-way Analysis of Variance (ANOVA) was conducted using EXPERTise cluster as the independent variable, and months of driving experience as the dependent variable. The length of driving experience reported by participants in the lesser cue utilization cluster (*M* = 38.24, *SD* = 12.69) did not differ significantly from those participants with greater levels of cue utilization (*M* = 39.50, *SD* = 19.70), *F*(1,57) = 0.088, *p* = 0.77, suggesting that assessments of cue utilization were not related to driving exposure.

#### Cue Utilization and Rail Task Performance

The primary aim of the present study was to establish whether differences existed between levels of cue utilization (cue typologies) and response latency across these four rail-control task blocks (a time block × cue typology interaction). A 2 × 4 mixed ANOVA, comprising two levels of cue utilization (greater and lesser) as a between-groups factor and four blocks of trials as a within-groups variable failed to reveal a statistically significant interaction between the variables, *F*(2.62,146.56) = 1.09, *p* = 0.349, $\eta_{p}^{2}$ = 0.019. This suggests that the changes evident in the mean response latency over trials occurred at similar rates, irrespective of cue utilization typology.

**FIGURE 2:**
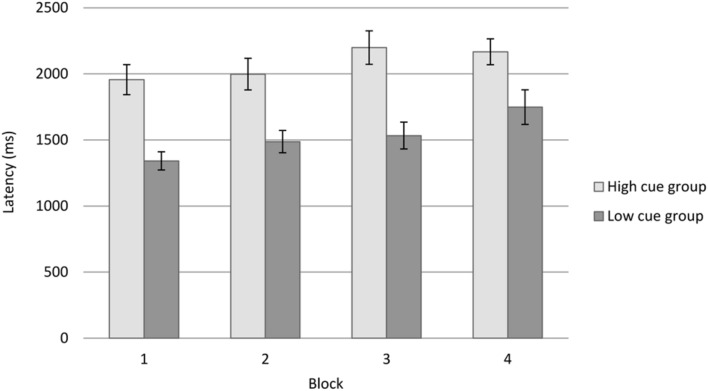
**Rail task response latencies by cue utilization typology and block number for Experiment 1.** Error bars represent ±1 *SE*.

Despite the fact that an interaction was not evident between cue utilization typology and blocks of trials, main effects were, nevertheless, evident for cue utilization typology, *F*(1,56) = 20.36, *p* < 0.001, *η*^2^ = 0.267 and for blocks of trials, *F*(2.60,147.89) = 7.37, *p* = 0.001, *η*^2^ = 0.114. Inspection of the mean response latencies (**Figure 2**) indicated that participants with a greater level of cue utilization recorded a *slower* mean response latency (*M* = 2079.70, *SD* = 395.67, *SE* = 80.77) across the four blocks of the rail-control task, in comparison to participants with a lesser level of cue utilization (*M* = 1527.36, *SD* = 498.59, *SE* = 85.51). Since there were no differences in the accuracy of the two groups, it suggests that participants with greater levels of cue utilization either withdrew cognitive resources to reduce the demand on cognitive load, or alternatively, invested cognitive resources to maintain accuracy.

*Post-hoc* analysis of the mean response latencies for blocks of trials indicated that mean response latencies in the first block of trials (*M* = 1595.51, *SD* = 558.33, *SE* = 73.31) were significantly lower than the fourth block (*M* = 1921.37, *SD* = 687.93, *SE* = 90.33), *t*(57) = –3.87, *p* < 0.001. This increase in mean response latency over time, despite no changes in task requirements, is consistent with the vigilance decrement.

### Discussion

This study was designed to examine whether, in response to a novel, short vigilance task, participants with a greater capacity for cue acquisition would adopt a strategy that would reduce the demands on cognitive resources. It was hypothesized that a strategy of least cognitive effort would be evident in an interaction that would emerge as the train control task progressed. On the basis of the Resource Theory explanation of the vigilance decrement, it was assumed that the increase in cognitive load that is associated with an extended period of watch would differentially affect those participants with lesser levels of cue utilization. Although a main effect was evident with progressive increases in response latency across blocks of trials, consistent with the hypothesized vigilance decrement, no statistically significant interaction occurred.

A main effect of cue utilization was also evident in which participants with a greater level of cue utilization showed increased response latencies in response to the diversion of trains. These mean response latencies were not associated with either cognitive ability (SPM scores) nor cognitive style (GEFT scores). However, it was unclear whether this response resulted in a reduction in cognitive load. Since there were no differences in the accuracy of responses amongst the two groups, the results suggest that participants with greater levels of cue utilization recognized that time was available in which to initiate a response to reroute misrouted trains, and adopted a strategy of least cognitive effort.

Although greater levels of cue utilization are normally associated with a reduction in response latency, this is not always the case. For example, in self-paced, targeting tasks such as rifle shooting and basketball (free throwing), superior shot accuracy is associated with longer quiet eye periods (the final fixation on the target prior to the initiation of movement) (Vickers, 1996; Vickers and Williams, 2007). As a result, skilled players tend to take more time to execute shots than lesser skilled players (Williams et al., 2002; Vickers, 2007). This suggests that the advantage afforded by greater levels of cue utilization lies in the capacity to recognize the need to adapt to different task demands. In the present study, there was no loss of performance associated with the increased response latency and it may have constituted a strategy of least cognitive effort which enabled the maintenance of performance despite the increase in cognitive demands.

There are at least two explanations for the lack of an interaction between levels of cue utilization and blocks of trials, the first of which relates to the hypothesized reduction in cognitive load. In particular, the self-pacing of one’s actions and responses within a task or job has been identified as a workload management strategy that effectively increases task control and reduces cognitive demands and anxiety (Johansson, 1981; Salvendy and Smith, 1981; Scerbo et al., 1993). However, it may be the case that the workload demands in the present study were insufficient to draw on the cognitive resources that would have been necessary to differentiate participants with greater or lesser levels of cue utilization.

An alternative explanation for the lack of an interaction relates to a potential investment of cognitive resources amongst participants with greater levels of cue utilization. Specifically, it might be argued that greater attention to the task, although overcompensating for the resources necessary to maintain accuracy, resulted in the increase in response latency. Experiment 2 was designed to differentiate the two explanations through the imposition of a secondary task that explicitly increased the cognitive demands during the rail control simulation.

## Experiment 2

Consistent with Experiment 1, participants in Experiment 2 completed the EXPERTise 1.0 situation judgment test and the 20-min simulated rail-control task. However, in addition to monitoring the rail display and re-routing trains as necessary, participants in Experiment 2 were asked to complete a secondary task during the final two blocks (10-min) of trials that comprised the monitoring task. This secondary task was designed to impose an explicit cognitive load, and required individuals to note the assigned number of each train (i.e., 888), together with the time at which it appeared (i.e., 2.07 PM).

Assuming that the advantage afforded by greater levels of cue utilization during the performance of a novel task is a reduction in cognitive load, it was anticipated that the imposition of a secondary task would impact the performance of participants with greater or lesser levels of cue utilization differently and at different stages of the task. It was hypothesized that an interaction would be evident in which participants with lesser levels of cue utilization would record an increase in response latency, while no effect would be evident for participants with greater levels of cue utilization.

### Method

#### Participants

Fifty-nine university students (15 males and 44 females) aged between 18 and 22 years (*M* = 18.81, *SD* = 1.06) participated in the study and received course credit for their participation. As in Experiment 1, individuals were excluded if they were not existing drivers, had acquired experience in the context of rail control, or were outside of the 18–22 year-old inclusion range. Participants in Experiment 1 of the study were also excluded from participating in Experiment 2.

#### Instruments

##### EXPERTise

The same four driving EXPERTise tasks (Wiggins et al., 2010) utilized in Experiment 1, were included as a composite measure of driving-related cue utilization across four cue-based problem solving and processing dimensions. An additional *Feature Identification* task was included, which exposed participants to a series of 18 different road images (photographs), each displayed for 500 ms, and required participants to estimate the speed limit of each road from four multiple-choice options (50–60, 70–80, 90–100 or 110+ km/hr). Designed to assess the capacity to rapidly extract key information from a driving-related scene and form an accurate judgment, a greater number of accurate judgments in this task was expected to reflect greater levels of cue utilization.

##### Rail control task

Participants in Experiment 2 completed the simulated train control task that was used in Experiment 1. However, in Experiment 2, participants completed the final two, 5-min blocks in conjunction with a secondary task.

##### Secondary task

A manipulation check was undertaken with five volunteers to ensure that the secondary task reduced the decision-time afforded to participants in the rail task, but did not induce an extremely low or an impossibly high level of workload such that the accuracy of responses would be impacted. The secondary task required participants to write down the train number and the time at which each train appeared on the screen. Following a 5-min period of familiarization, three volunteers completed the first half of the rail task (10-min) with the inclusion of the secondary task, while two volunteers completed the second half of the rail task (10-min) with the inclusion of the secondary task. Trials were counterbalanced to control for sequencing effects, such as fatigue, that were unrelated to the secondary task. The manipulation check revealed no errors in the secondary task (all trains were correctly logged), while response latency was greater for the dual task condition (*M* = 3063 ms) compared to the vigil-only condition (*M* = 2691 ms) suggesting that the secondary task increased the workload to an adequate but not extreme degree.

##### Subjective workload

Subjective workload was measured by the NASA Task Load Index (NASA-TLX: Hart and Staveland, 1988), a widely used and validated multi-dimensional rating procedure that provides an overall workload score based on a weighted average of ratings on six subscales: Mental demands, physical demands, temporal demands, performance, effort, and frustration (Hart and Staveland, 1988; Xiao et al., 2005) on a scale of 1–100. Participants completed the NASA-TLX following the single rail-task condition (Blocks 1 and 2) and again following the secondary task condition (Blocks 3 and 4).

#### Procedure

As in Experiment 1, participants were tested individually and completed the study in sessions of 90 min. Following the completion of a demographic questionnaire, participants undertook the EXPERTise tasks and a 5-min practice trial to orient participants to the rail task. Prior to the rail control task, instructions were provided to participants in relation to the distractor task and they were given the paper-based secondary-task sheet. Once participants indicated that the instructions were understood, the simulated rail control task commenced. After 10 minutes, the rail task was paused by the researcher and participants completed the NASA-TLX. The rail task then recommenced, and for the remaining ten minutes of the task, participants diverted trains and completed the secondary-task sheet concurrently. Following the completion of the rail task, participants again completed the NASA-TLX.

### Results

#### Cue Utilization Typologies

Consistent with Experiment 1, a cluster analysis was undertaken using aggregated EXPERTise *z* scores for all five tasks to identify the cue utilization typologies that corresponded with relatively greater and lesser levels of cue utilization. Two groups were identified with centroids corresponding to higher variance in the Paired Association and Feature Discrimination tasks, lower response latency in the Feature Identification tasks (reversed *z* scores), and lower ratio of sequential selections in the Information Acquisition task (reversed *z* score). In this case, the cluster analysis (**Table 2**) classified 22 participants in the lower cue utilization typology (cluster 1) and 33 participants in the higher cue utilization typology (cluster 2).

**Table 2:** Cluster centroids for the EXPERTise task scores.

	Cluster 1 (*n* = 22)	Cluster 2 (*n* = 33)
Paired association	–0.83	0.56
Feature discrimination	–0.84	0.53
Feature identification	–0.30	0.21
Feature identification II	–0.45	0.33
Information acquisition	–0.18	0.18

#### Driving Experience and Cue Utilization

Consistent with Experiment 1, the duration of driving experience (months) reported by participants in the lesser cue utilization cluster (*M* = 29.73, *SD* = 13.06) did not differ significantly from those participants who were classified in the greater cue utilization cluster (*M* = 29.57, *SD* = 13.60), *F*(1,50) = 0.002, *p* = 0.97. This suggests that differences in cue utilization did not result from differences in participants’ driving experience.

#### Rail Task Performance

Consistent with the results in Experiment 1, a floor effect was evident for the frequency of errors during the rail control task (range = 0–4, *Mdn* = 1) with 68% of participants committing either zero or a single error during exposure to 67 trains. A Chi-square test of independence indicated there were no significant differences in the distribution of errors across the four time intervals, χ2 (3,59) = 5.78, *p* = 0.123. The frequency of errors committed was unrelated to response latencies (Spearman’s non-parametric, 0.18 ≤ *r* ≤ 0.26, *p* > 0.05).

#### Cue Utilization and Rail Task Latencies

**FIGURE 3:**
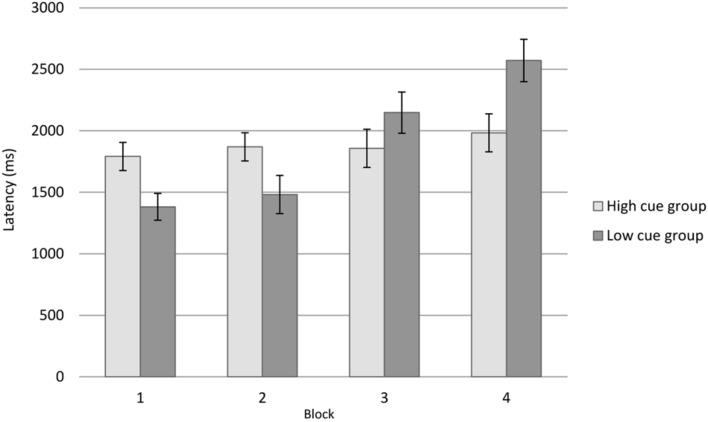
**Rail task response latencies by cue utilization typology and block number for Experiment 2.** Error bars represent ±1 *SE*.

To investigate whether the imposition of the secondary task had a greater impact on participants with lesser levels of cue utilization compared to those participants with greater levels, a 2 × 4 mixed repeated ANOVA was undertaken, including the two levels of cue utilization (greater, lesser) as a between-groups variable and the four blocks of trials as a within groups variable. Consistent with the hypothesis, an interaction was evident between cue utilization and block trials, *F*(1.80,90.21) = 10.81, *p* < 0.001, $\eta_{p}^{2}$ = 0.178 (Greenhouse–Geisser correction), in which the mean response latency for participants increased with lesser levels of cue utilization, while the mean response latency for participants with greater levels of cue utilization remained relatively consistent (**Figure 3**). This suggests that the imposition of the secondary task had a greater impact on participants with lesser levels of cue utilization in comparison to participants with greater levels of cue utilization.

A main effect was evident for blocks of trials, *F*(1.65,95.72) = 12.11, *p* < 0.001, $\eta_{p}^{2}$ = 0.173. *Post hoc* analysis of the mean response latencies for blocks of trials indicated that the mean response latencies in the first block of trials (*M* = 1608.56, *SD* = 594.66, *SE* = 77.42) were significantly lower than in the final block of trials (*M* = 2226.61, *SD* = 851.81, *SE* = 110.90), *t*(58) = –4.51, *p* < 0.00. The main effect of cue utilization was not statistically significant, *F*(1,50) = 0.17, *p* = 0.90.

As is evident from **Figure 3**, the pattern of response latencies following the imposition of the secondary task differed on the basis of levels of cue utilization. This suggests that the relative impact of the secondary task was greatest for participants with lesser levels of cue utilization than was the case for participants with greater levels of cue utilization.

#### Cue Utilization and Mental Workload Perceptions

To investigate whether the imposition of the secondary task impacted participants’ perceptions of mental workload, a 2 × 2 mixed repeated ANOVA was undertaken, with cue utilization level (greater and lesser) as the between-groups factor and TLX scores (single-condition and dual-condition) as the within-groups variable. The results revealed a statistically significant main effect for perceptions of mental workload, *F*(1,50) = 85.33, *p* < 0.001, $\eta_{p}^{2}$ = 0.631, in which participants perceived the task workload in the dual condition as significantly greater (*M* = 26.83, *SD* = 1.90), than during the single task condition (*M* = 14.78, *SD* = 1.40), *t*(58) = –9.22, *p* < 0.001. There was no main effect for cue utilization, *F*(1,50) = 0.58, *p* = 0.449.

**FIGURE 4:**
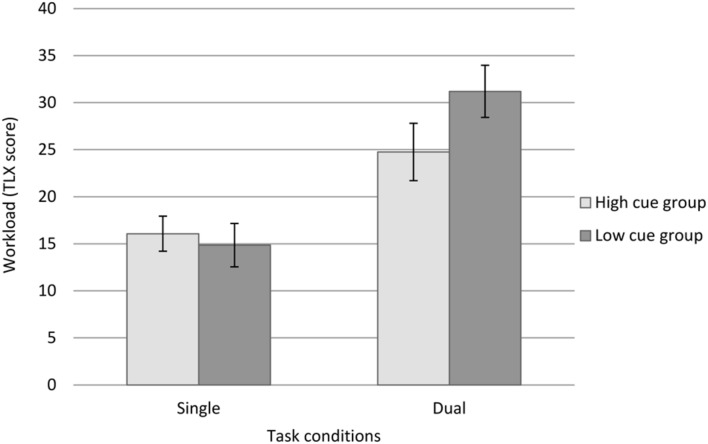
**Mental workload across task conditions, by cue utilization typology.** Error bars represent ±1 *SE*.

Consistent with the results pertaining to response latency, a statistically significant interaction was evident between perceptions of mental workload and cue utilization, *F*(1,50) = 8.00, *p* = 0.007, $\eta_{p}^{2}$ = 0.138. As is evident from **Figure 4**, the pattern of perceived mental workload (as measured by the NASA-TLX) following the imposition of the secondary task differed on the basis of levels of cue utilization. Specifically, the perceived impact of the secondary task was greatest for participants with lesser levels of cue utilization.

### Discussion

The introduction of the secondary task part-way during the 20-min period of rail control was designed to impose an explicit cognitive demand on the performance of participants. It was reasoned that if participants with greater levels of cue utilization had adopted a strategy that effectively reduced the demands on cognitive resources, then an interaction should be evident following the introduction of the secondary task during the final two, 5-min blocks of the 20-min trial. Specifically, it was hypothesized that participants with lesser levels of cue utilization would record an increase in response latency, while only a minimal effect would be evident for participants with greater levels of cue utilization. Consistent with the hypothesis, mean response latencies for participants with lesser levels of cue utilization increased following the introduction of the secondary task and continued to increase as the task progressed, while the mean response latencies for participants with greater levels of cue utilization remained consistent with the vigilance decrement that was evident in Experiment 1. This effect occurred independent of driving experience but was reflected in perceptions of mental workload.

## General Discussion

In response to a novel task, the rapid development of associational cues in memory is one means by which the cognitive demands of a task can be minimized (Norman and Shallice, 1986; Chung and Byrne, 2008; Evans, 2008). The aim of the research presented in this paper was to examine whether differences in cue utilization were associated with differences in performance during a novel, simulated rail control task, and whether these differences in performance reflected a reduction in cognitive load. On the assumption that cognitive load increases with sustained attention to a task (Helton et al., 2005; Helton and Warm, 2008), it was anticipated that individuals with relatively greater levels of cue utilization would be relatively less impacted by the sustained attentional demands imposed by a simulated rail-control task in which participants were asked to identify and correct the path of trains that had periodically been misrouted.

Two experiments were conducted with motor vehicle drivers aged between 18 and 22 years who undertook an assessment of cue utilization using the driving battery of EXPERTise 1.0. In Experiment 1, participants who were identified a priori with a relatively greater level of cue utilization on the basis of their scores on EXPERTise 1.0, recorded a mean response latency greater than that recorded by participants with relatively lesser levels of cue utilization. The effect remained consistent across the four blocks of 5-min trials within the rail-control task. Importantly, there were no differences in accuracy and, in fact, a floor effect was evident in relation to errors.

A vigilance decrement was evident in the increases in response latency recorded across blocks of trials, irrespective of participants’ level of cue utilization. This suggests that, although an increase in cognitive load may have been associated with sustained attention to the task, the level was insufficient to differentiate the performance of participants on the basis of their cue utilization. Consequently, Experiment 2 adopted a similar methodology but included a secondary task to invoke an explicit cognitive load part-way through the simulated rail control task.

The performance of participants in Experiment 2 during the initial two blocks of trials appeared consistent with the results from Experiment 1, whereby the response latency recorded was higher for participants with greater levels of cue utilization. However, once the secondary task was initiated, the response latency of participants with lesser levels of cue utilization increased, while the response latency amongst participants with greater levels of cue utilization remained relatively consistent. This suggests that the relative impact of the secondary task was greater for participants with lesser levels of cue utilization than it was for participants with greater levels of cue utilization.

The relative consistency of response latencies recorded for participants with higher levels of cue utilization across all blocks despite the imposition of a secondary task, suggests that they had adopted a strategy that reduced the demands on cognitive load. Until the introduction of a secondary task, the mean response latency for participants with greater levels of cue utilization was consistently greater than the mean response latency recorded by participants with lesser levels of cue utilization. Therefore, it might be concluded that participants were adopting a strategy of self-pacing, which effectively increased task control and reduced cognitive demands (Johansson, 1981; Salvendy and Smith, 1981; Scerbo et al., 1993). As a decision to re-route trains in the rail simulation task could be initiated up to seven seconds from the appearance of a train, those participants with greater levels of cue utilization appear to have recognized this opportunity and utilized the additional time, without sacrificing accuracy.

In contrast, the pattern of results for those participants with lesser levels of cue utilization, suggests that, until the imposition of the secondary task, these participants may have been responding rapidly and reactively, rather than in a manner consistent with the strategic conservation of resources to manage workload (Hollnagel, 2002; Hollnagel and Woods, 2005; Loft et al., 2007). Their rapid increase in mean response latencies subsequent to the imposition of the secondary task suggested that their reactive responses were unable to be sustained with the increasing level of workload.

It is noteworthy, however, that those participants with lesser levels of cue utilization maintained consistent (and low) levels of error rates throughout the rail task, and this occurred despite the increased workload imposed by the secondary task. Therefore, it is also possible that those participants with lesser levels of cue utilization may have adopted a strategy that increasingly sacrificed speed for accuracy. Given that the workload of the task imposed demands that did not impact accuracy, it is likely that a further increase in cognitive demands would, despite efforts to minimize effort, exhaust the information processing resources of those participants with lesser levels of cue utilization and result in a deterioration in accuracy. To explore if this is the case, future research may consider increasing the level of cognitive demand by either extending the duration of the vigil (e.g., Freeman et al., 2004; Nelson et al., 2014) or increasing the demands of the task (Matthews and Davies, 1998; Smit et al., 2004) to a point where accuracy is impeded (Smit et al., 2004).

Overall, the results of both experiments provide support for the assertion that a relatively greater capacity for cue utilization is associated with an increased capacity to cope with the demands of a novel task. Throughout both experiments, several control measures were utilized to ensure that performance differences between individuals with lesser and greater levels of cue utilization were not due to cognitive ability nor cognitive style. These variables were not related to response latencies. Consistent with previous research (Smeeton et al., 2004; Müller and Abernethy, 2012; Moore and Müller, 2014; Wiggins et al., 2014), our results suggest that a propensity to identify critical cues and rapidly establish feature-event relationships may provide an opportunity to reduce cognitive demands, thereby enabling the acquisition of new features and/or the opportunity to revise or refine existing features.

In practice, implications that arise from the present study present tangible opportunities in the context of selection and training. The ability to identify the levels of cue utilization may provide the basis to differentiate job applicants that are more or less likely to acquire skills in the absence of a dedicated training regime. The outcomes might also be applied to identify employees who are most in need of a training intervention, particularly in the context of the identification of key features that might enable a reduction in cognitive load and the subsequent acquisition and revision of feature-event relationships in the form of cues (Wulf et al., 2000; Lagnado et al., 2006).

What remains to be established is the extent to which the association between cue utilization and performance evident in the present research can be generalized. For example, driving and rail control both involve visual perception and spatial skills. The driving version of EXPERTise may be less capable of differentiating performance beyond this context. It is also noteworthy that while the results of this study suggest that participants with a greater capacity for cue utilization adopted a strategy that minimized the impact of additional cognitive load on their performance, the precise nature of that strategy (which may pertain to the utilization of available time to self-pace) has yet to be investigated and explicated.

## Conclusion

The present study was designed to examine whether differences in cue utilization were associated with differences in performance during a novel, simulated rail control task, and whether these differences in performance reflected a reduction in cognitive load. The results of two experiments suggested that levels of cue utilization were associated with differences in response latencies throughout the simulated rail task, and that individuals with a greater level of cue utilization were able to adopt a strategy that effectively reduced cognitive load without sacrificing accuracy.

## Author Contributions

All authors listed, have made substantial, direct and intellectual contribution to the work, and approved it for publication.

## Conflict of Interest Statement

The authors declare that the research was conducted in the absence of any commercial or financial relationships that could be construed as a potential conflict of interest.

## References

[B1] Abernethy B. (1987). Anticipation in sport: a review. *Phys. Educ. Rev.* 10 5–16.

[B2] Abernethy B. (1990). Anticipation in squash: differences in advance cue utilization between expert and novice players. *J. Sports Sci.* 8 17–34. 10.1080/02640419008732128 2359149

[B3] Ackerman B. P. Rathburn J. (1984). The effect of recognition experience on cued recall in children and adults. *Child Dev.* 55 1855–1864. 10.2307/1129932

[B4] Ackerman P. L. (1986). Individual differences in information processing: an investigation of intellectual abilities and task performance during practice. *Intelligence* 10 101–139. 10.1016/0160-2896(86)90010-3

[B5] Ackerman P. L. (2007). New developments in understanding skilled performance. *Curr. Dir. Psychol. Res.* 16 235–239. 10.1111/j.1467-8721.2007.00511.x

[B6] Ackerman P. L. Beier M. E. (2007). Further explorations of perceptual speed abilities, in the context of assessment methods, cognitive abilities and individual differences during skill acquisition. *J. Exp. Psychol.* 13 249–272. 10.1037/1076-898X.13.4.249 18194049

[B7] Austin E. J. (2005). Emotional intelligence and emotional information processing. *Pers. Individ. Differ.* 39 403–414. 10.1016/j.paid.2005.01.017

[B8] Beilock S. L. Bertenthal B. I. McCoy A. M. Carr T. H. (2004). Haste does not always make waste: expertise, direction of attention, and speed versus accuracy in performing sensorimotor skills. *Psychon. Bull. Rev.* 11 373–379. 10.3758/BF03196585 15260208

[B9] Bellenkes A. H. Wickens C. D. Kramer A. F. (1997). Visual scanning and pilot expertise: the role of attentional flexibility and mental model development. *Aviat. Space Environ. Med.* 68 569–579. 9215461

[B10] Berkenkötter K. Hannemann U. (2006). “Modeling the railway control domain rigorously with a UML 2.0 profile,” in *Computer Safety, Reliability, and Security*, eds Saglietti F. Oster N. (Berlin: Springer), 398–411.

[B11] Brunswik E. (1955). Representative design and probabilistic theory in a functional psychology. *Psychol. Rev.* 62 193–271. 10.1037/h0047470 14371898

[B12] Caffarra P. Vezzadini G. Zonato F. Copelli S. Venneri A. (2003). A normative study of a shorter version of Raven’s progressive matrices 1938. *Neurol. Sci.* 24 336–339. 10.1007/s10072-003-0185-0 14716529

[B13] Cegarra J. Hoc J. M. (2006). Cognitive styles as an explanation of experts’ individual differences: a case study in computer-assisted troubleshooting diagnosis. *Int. J. Hum. Comput. Stud.* 64 123–136. 10.1016/j.ijhcs.2005.06.003

[B14] Chung P. H. Byrne M. D. (2008). Cue effectiveness in mitigating post completion errors in a routine procedural task. *Int. J. Hum. Comput. Stud.* 66 217–232. 10.1016/j.ijhcs.2007.09.001

[B15] Conway A. R. Cowan N. Bunting M. F. Therriault D. J. Minkoff S. R. (2002). A latent variable analysis of working memory capacity, short-term memory capacity, processing speed, and general fluid intelligence. *Intelligence* 30 163–183. 10.1016/S0160-2896(01)00096-4

[B16] Elander J. West R. French D. (1993). Behavioral correlates of individual differences in road-traffic crash risk: an examination of methods and findings. *Psychol. Bull.* 113 279–294. 10.1037/0033-2909.113.2.279 8451335

[B17] Ericsson K. A. Lehmann A. C. (1996). Expert and exceptional performance: evidence of maximal adaptation to task constraints. *Annu. Rev. Psychol.* 47 273–305. 10.1146/annurev.psych.47.1.273 15012483

[B18] Evans J. S. B. (2008). Dual-processes accounts of reasoning. *Annu. Rev. Psychol.* 59 255–278. 10.1146/annurev.psych.59.103006.093629 18154502

[B19] Farrington-Darby T. Wilson J. R. Norris B. J. Clarke T. (2006). A naturalistic study of railway controllers. *Ergonomics* 49 1370–1394. 10.1080/00140130600613000 17008261

[B20] Freeman F. G. Mikulka P. J. Scerbo M. W. Scott L. (2004). An evaluation of an adaptive automation system using a cognitive vigilance task. *Biol. Psychol.* 67 283–297. 10.1016/j.biopsycho.2004.01.002 15294387

[B21] Hart S. G. Staveland L. E. (1988). “Development of NASA-TLX (Task Load Index): results of empirical and theoretical research,” in *Human Mental Workload*, eds Hancock P. A. Meshkati N. (Amsterdam: North-Holland), 139–183.

[B22] Helton W. S. Hollander T. D. Warm J. S. Matthews G. Dember W. N. Wallaart M. (2005). Signal regularity and the mindlessness model of vigilance. *Br. J. Psychol.* 96 249–261. 10.1348/000712605X38369 15969834

[B23] Helton W. S. Warm J. S. (2008). Signal salience and the mindlessness theory of vigilance. *Acta Psychol.* 129 18–25. 10.1016/j.actpsy.2008.04.002 18499079

[B24] Ho T. K. Mao B. H. Yuan Z. Z. Liu H. D. Fung Y. F. (2002). Computer simulation and modeling in railway applications. *Comput. Phys. Commun.* 143 1–10. 10.1016/S0010-4655(01)00410-6

[B25] Hollnagel E. (2002). Time and time again. *Theor. Issues Ergon. Sci.* 3 143–158. 10.1080/14639220210124111

[B26] Hollnagel E. Woods D. D. (2005). *Joint Cognitive Systems: Foundations of Cognitive Systems Engineering.* Boca Raton, FL: CRC Press.

[B27] Hull C. L. (1943). *Principles of Behavior.* New York: Appleton-Century-Crofts.

[B28] Jackson R. C. Mogan P. (2007). Advance visual information, awareness, and anticipation skill. *J. Mot. Behav.* 39 341–351. 10.3200/JMBR.39.5.341-352 17827112

[B29] Jaeggi S. M. Buschkuehl M. Jonides J. Shah P. (2011). Short- and long-term benefits of cognitive training. *Proc. Natl. Acad. Sci. U.S.A.* 108 10081–10086. 10.1073/pnas.1103228108 21670271 PMC3121868

[B30] Johansson G. (1981). “Psychoneuroendocrine correlates of unpaced and paced performance,” in *Machine Pacing and Occupational Stress*, eds Salvendy G. Smith M. J. (London: Taylor & Francis), 277–286.

[B31] Kane M. J. Engle R. W. (2003). Working-memory capacity and the control of attention: the contributions of goal neglect, response competition, and task set to Stroop interference. *J. Exp. Psychol.* 132 47–70. 10.1037/0096-3445.132.1.47 12656297

[B32] Kaplan R. M. Saccuzzo D. P. (2008). *Psychological Testing: Principles, Applications, and Issues*, 7th Edn Belmont, CA: Wadsworth Thomson Learning.

[B33] Kepner M. D. Neimark E. D. (1984). Test–retest reliability and differential patterns of score change on the Group Embedded Figures Test. *J. Pers. Soc. Psychol.* 46 1405–1413. 10.1037/0022-3514.46.6.1405 6737219

[B34] Klein G. (2011). “Intuition and naturalistic decision-making,” in *Handbook of Intuition Research*, ed. Sinclair M. (Cheltenham: Edward Elgar Publishing), 69–78.

[B35] Klein G. A. Calderwood R. Clinton-Cirocco A. (1986). Rapid decision making on the fire ground. *Hum. Fact. Ergon. Soc. Ann. Meet. Proc.* 30 576–580. 10.1177/154193128603000616

[B36] Kool W. McGuire J. T. Rosen Z. B. Botvinick M. M. (2010). Decision making and the avoidance of cognitive demand. *J. Exp. Psychol.* 139 665–682. 10.1037/a0020198 PMC2970648 20853993

[B37] Lagnado D. A. Newell B. R. Kahan S. Shanks D. R. (2006). Insight and strategy in multiple-cue learning. *J. Exp. Psychol.* 135 162–183. 10.1037/0096-3445.135.2.162 16719649

[B38] Leach J. Morris P. E. (1998). Cognitive factors in the close visual and magnetic particle inspection of welds underwater. *Hum. Fact.* 40 187–197. 10.1518/001872098779480460 9720456

[B39] Lenior T. M. J. (1993). Analyses of cognitive processes in train traffic control. *Ergonomics* 36 1361–1368. 10.1080/00140139308968005 8262029

[B40] Loft S. Sanderson P. Neal A. Mooij M. (2007). Modeling and predicting mental workload in en route air traffic control: critical review and broader implications. *Hum. Factors* 49 376–399. 10.1518/001872007X197017 17552304

[B41] Loveday T. Wiggins M. W. Festa M. Schell D. Twigg D. (2013a). “Pattern recognition as an indicator of diagnostic expertise,” in *Pattern Recognition - Applications and Methods* Vol. 204 eds Latorre Carmona P. Sanchez J. S. Fred A. L. N. (New York, NY: Springer-Verlag), 1–11.

[B42] Loveday T. Wiggins M. W. Harris J. M. O’Hare D. Smith N. (2013b). An objective approach to identifying diagnostic expertise among power system controllers. *Hum. Fact.* 55 90–107. 10.1177/0018720812450911 23516796

[B43] Loveday T. Wiggins M. W. Searle B. J. Festa M. Schell D. (2013c). The capability of static and dynamic features to distinguish competent from genuinely expert practitioners in pediatric diagnosis. *Hum. Fact.* 55 125–137. 10.1177/0018720812448475 23516798

[B44] Loveday T. Wiggins M. W. Searle B. J. (2014). Cue utilization and broad indicators of workplace expertise. *J. Cogn. Eng. Decis. Mak.* 8 98–113. 10.1177/1555343413497019

[B45] Matthews G. Davies D. R. (1998). “Arousal and vigilance: the role of task factors,” in *Viewing Psychology as a Whole: The Integrative Science of William N. Dember*, eds Hoffman R. B. Sherrick M. F. Warm J. S. (Washington, DC: American Psychological Association).

[B46] Moore C. G. Müller S. (2014). Transfer of expert visual anticipation to a similar domain. *Q. J. Exp. Psychol.* 67 186–196. 10.1080/17470218.2013.798003 23700967

[B47] Morrison B. Wiggins M. W. Bond N. Tyler M. (2013). Measuring cue strength as a means of identifying an inventory of expert offender profiling cues. *J. Cogn. Eng. Decis. Mak.* 7 211–226. 10.1177/1555343412459192

[B48] Moutafi J. Furnham A. Tsaousis I. (2006). Is the relationship between intelligence and trait neuroticism mediated by test anxiety? *Pers. Individ. Differ.* 40 587–597. 10.1016/j.paid.2005.08.004

[B49] Müller S. Abernethy B. (2012). Expert anticipatory skill in striking sports: a review and a model. *Res. Q. Exerc. Sport* 83 175–187. 10.1080/02701367.2012.10599848 22808703

[B50] Müller S. Abernethy B. Farrow D. (2006). How do world-class cricket batsmen anticipate a bowler’s intention? *Q. J. Exp. Psychol.* 59 2162–2186. 10.1080/02643290600576595 17095494

[B51] Neerincx M. A. de Greef H. P. (1998). Cognitive support: extending human knowledge and processing capacities. *Hum. Comput. Interact.* 13 73–106. 10.1207/s15327051hci1301_3

[B52] Nelson J. T. McKinley R. A. Golob E. J. Warm J. S. Parasuraman R. (2014). Enhancing vigilance in operators with prefrontal cortex transcranial direct current stimulation (tDCS). *Neuroimage* 85 909–917. 10.1016/j.neuroimage.2012.11.061 23235272

[B53] Norman D. A. Shallice T. (1986). “Attention to action: willed and automatic control of behavior,” in *Consciousness and Self-Regulation: Advances in Research* Vol. 4 eds Davidson R. J. Schwartz G. E. Shapiro D. (New York, NY: Plenum), 1–17.

[B54] Oltman P. Raskin E. Witkin H. (2003). *Group Embedded Figures Test.* California, CA: Mind Garden, Inc.

[B55] Pauley K. O’Hare D. Wiggins M. (2009). Measuring expertise in weather-related aeronautical risk perception: the validity of the Cochran–Weiss–Shanteau (CWS) Index. *Int. J. Aviat. Psychol.* 19 201–216. 10.1080/10508410902979993

[B56] Perry N. C. Wiggins M. W. Childs M. Fogarty G. (2013). The application of reduced-processing decision support systems to facilitate the acquisition of decision-making skills. *Hum. Fact.* 55 535–544. 10.1177/0018720812467367 23829028

[B57] Raven J. Raven J. C. Court J. H. (1998). *Manual for Raven’s Progressive Matrices and Vocabulary Scales. Section* 1 General Overview San Antonio, TX: Harcourt Assessment.

[B58] Raven J. Raven J. C. Court J. H. (2000). *Manual for Raven’s Progressive Matrices and Vocabulary Scales. Section* 3: *The Standard Progressive Matrices*. San Antonio, TX: Harcourt Assessment.

[B59] Rose C. L. Murphy L. B. Byard L. Nikzad K. (2002). The role of the Big Five personality factors in vigilance performance and workload. *Eur. J. Pers.* 16 185–200. 10.1002/per.451

[B60] Salthouse T. A. (1991). “Expertise as the circumvention of human information processing,” in *Toward a General Theory of Expertise: Prospects and Limits*, eds Ericsson K. A. Smith J. (Cambridge, NY: Cambridge University Press), 286–300.

[B61] Salvendy G. Smith M. J. (eds) (1981). *Machine Pacing and Occupational Stress.* London: Taylor & Francis.

[B62] Scerbo M. W. Greenwald C. Q. Sawin D. A. (1993). The effects of subject-controlled pacing and task type on sustained attention and subjective workload. *J. Gen. Psychol.* 120 293–307. 10.1080/00221309.1993.9711149

[B63] Schriver A. T. Morrow D. G. Wickens C. D. Talleur D. A. (2008). Expertise differences in attentional strategies related to pilot decision making. *Hum. Fact.* 50 864–878. 10.1518/001872008X374974 19292010

[B64] Schvaneveldt R. W. Beringer D. B. Lamonica J. A. (2001). Priority and organization of information accessed by pilots in various phases of flight. *Int. J. Aviat. Psychol.* 11 253–280. 10.1207/S15327108IJAP1103_02

[B65] Schyns P. G. (1998). Diagnostic recognition: task constraints, object information, and their interactions. *Cognition* 67 147–179. 10.1016/S0010-0277(98)00016-X 9735539

[B66] Simonton D. K. (2007). Talent “and” expertise: the empirical evidence for genetic endowment. *High Ability Stud.* 18 83–84. 10.1080/13598130701350890

[B67] Simonton D. K. (2008). Scientific talent, training, and performance: intellect, personality, and genetic endowment. *Rev. Gen. Psychol.* 12 28–46. 10.1037/1089-2680.12.1.28

[B68] Singer R. N. Janelle C. M. (1999). Determining sport expertise: from genes to supremes. *Int. J. Sport Psychol.* 30 117–150.

[B69] Small A. J. Wiggins M. W. Loveday T. (2014). Cue-based processing capacity, cognitive load and the completion of simulated short-duration vigilance tasks in power transmission control. *Appl. Cogn. Psychol.* 28 481–487. 10.1002/acp.3016

[B70] Smeeton N. J. Ward P. Williams A. M. (2004). Do pattern recognition skills transfer across sports? A preliminary analysis. *J. Sports Sci.* 22 205–213. 10.1080/02640410310001641494 14998098

[B71] Smit A. S. Eling P. A. Coenen A. M. (2004). Mental effort causes vigilance decrease due to resource depletion. *Acta Psychol.* 115 35–42. 10.1016/j.actpsy.2003.11.001 14734240

[B72] Temple J. G. Warm J. S. Dember W. N. Jones K. S. LaGrange C. M. Matthews G. (2000). The effects of signal salience and caffeine on performance, workload, and stress in an abbreviated vigilance task. *Hum. Factors* 42 183–194. 10.1518/001872000779656480 11022879

[B73] Thompson C. P. Cowan T. M. Frieman J. (1993). *Memory Search by a Memorist.* Hillsdale, NJ: Lawrence Erlbaum Associates.

[B74] Vessey I. Galletta D. (1991). Cognitive fit: an empirical study of information acquisition. *Informat. Syst. Res.* 2 63–84. 10.1287/isre.2.1.63

[B75] Vickers J. N. (1996). Visual control while aiming at a far target. *J. Exp. Psychol.* 22 342–354. 10.1037//0096-1523.22.2.342 8934848

[B76] Vickers J. N. (2007). *Perception, Cognition, and Decision Training: The Quiet Eye in Action.* Champaign, IL: Human Kinetics.

[B77] Vickers J. N. Williams A. M. (2007). Performing under pressure: the effects of physiological arousal, cognitive anxiety, and gaze control in biathlon. *J. Motor Behav.* 39 381–394. 10.3200/JMBR.39.5.381-394 17827115

[B78] Weiss D. J. Shanteau J. (2003). Empirical assessment of expertise. *Hum. Fact.* 45 104–116. 10.1518/hfes.45.1.104.27233 12916584

[B79] Wiggins M. O’Hare D. (1995). Expertise in aeronautical weather-related decision making: a cross-sectional analysis of general aviation pilots. *J. Exp. Psychol.* 1 305–320.

[B80] Wiggins M. Stevens C. Howard A. Henley I. O’Hare D. (2002). Expert, intermediate and novice performance during simulated pre-flight decision making. *Austr. J. Psychol.* 54 162–167. 10.1080/00049530412331312744

[B81] Wiggins M. W. (2014). The role of cue utilisation and adaptive interface design in the management of skilled performance in operations control. *Theor. Issues Ergon. Sci.* 15 283–292. 10.1080/1463922X.2012.724725

[B82] Wiggins M. W. (2015). *Diagnostic Expertise in Organizational Environments.* Surrey: Ashgate Publishing.

[B83] Wiggins M. W. Brouwers S. Davies J. Loveday T. (2014). Trait-based cue utilization and initial skill acquisition: Implications for models of the progression to expertise. *Front. Psychol.* 5:541 10.3389/fpsyg.2014.00541 PMC4042495 24917844

[B84] Wiggins M. W. Harris J. Loveday T. O’Hare D. (2010). *EXPERT Intensive Skills Evaluation (EXPERTise) Test.* Sydney: Macquarie University.

[B85] Williams A. M. Singer R. N. Frehlich S. G. (2002). Quiet eye duration, expertise, and task complexity in near and far aiming tasks. *J. Mot. Behav.* 34 197–207. 10.1080/00222890209601941 12057892

[B86] Witkin H. A. (1976). “Cognitive style in academic performance and in teacher-student relations,” in *Individuality in Learning*, eds Messick S. (San Francisco, CA: Jossey-Bass), 38–72.

[B87] Witkin H. A. Oltman P. K. Raskin E. Karp S. A. (1971). *A Manual for the Embedded Figures Tests.* Palo Alto, CA: Consulting Psychologists Press.

[B88] Witkin H. A. Oltman P. K. Raskin E. Karp S. A. (2002). *Group Embedded Figures Test. Sampler Set. Manual and Sample Figures and Scoring.* Menlo Park, CA: Mind Garden Inc.

[B89] Wulf G. McNevin N. H. Fuchs T. Ritter F. Toole T. (2000). Attentional focus in complex skill learning. *Res. Q. Exerc. Sport* 71 229–239. 10.1080/02701367.2000.10608903 10999260

[B90] Xiao Y. M. Wang Z. M. Wang M. Z. Lan Y. J. (2005). The appraisal of reliability and validity of subjective workload assessment technique and NASA-Task Load Index. *Chin. J. Industr. Hygiene Occupat. Dis.* 23 178–181. 16124892

[B91] Zimmerman B. J. (2002). Becoming a self-regulated learner: an overview. *Theory Pract.* 41 64–70. 10.1207/s15430421tip4102_2

[B92] Zimmerman B. J. (2008). Investigating self-regulation and motivation: historical background, methodological developments, and future prospects. *Am. Educ. Res. J.* 45 166–183. 10.3102/0002831207312909

